# A chronic hemodialysis patient with isolated pulmonary valve infective endocarditis caused by non-*albicans Candida*: a rare case and literature review

**DOI:** 10.1186/s12882-017-0706-3

**Published:** 2017-09-06

**Authors:** Chih-Hao Chang, Myo-Ming Huang, Dong-Feng Yeih, Kuo-Cheng Lu, Yi-Chou Hou

**Affiliations:** 1 Department of Thoracic Medicine, Chang-Gang Memorial Hospital, Linkou Branch, No.5, Fuxing St., Guishan Dist, Taoyuan City, 33305 Taiwan, Republic of China; 2 Department of Internal Medicine, Cardinal Tien Hospital An-Kang branch, School of Medicine, Fu-Jen Catholic University, No. 15, Chezi Road., Hsin-Tien District, New Taipei City, 23155 Taiwan, Republic of China; 3 Department of Internal Medicine, Cardinal Tien Hospital, School of Medicine, Fu-Jen Catholic University, 362 Chung-Cheng Road, Hsin-Tien District, New Taipei City, 23148 Taiwan, Republic of China; 4 Graduate Institute of Clinical Medicine, College of Medicine, Taipei Medical University, Taipei, Taiwan, Republic of China; 5 Division of Nephrology, Department of Medicine, Tri-Service General Hospital, National Defense Medical Center, Taipei, Taiwan, Republic of China

**Keywords:** *Candida guilliermondii*, Pulmonary valve, Infective endocarditis

## Abstract

**Background:**

Isolated pulmonary valve infective endocarditis caused by *Candida* is rare in chronic hemodialysis patients. The 2009 Infectious Diseases Society of America guidelines suggest the combined use of surgery and antibiotics to treat candidiasis; however, successful nonsurgical treatment of *Candida* endocarditis has been reported.

**Case presentation:**

A 63-year-old woman with end-stage kidney disease was admitted to our hospital after experiencing disorientation for 5 days. The patient was permanently bedridden because of depression, and denied active intravenous drug use. She received maintenance hemodialysis through a tunneled-cuffed catheter. An initial blood culture grew *Candida guilliermondii* without other bacteria. Subsequent blood cultures and tip culture of tunneled-cuffed catheter also grew *C. guilliermondii*, even after caspofungin replaced fluconazole. A 1.2-cm mobile mass was observed on the pulmonary valve. Surgical intervention was suggested, but the family of the patient declined because of her multiple comorbidities. The patient was discharged with a prescription of fluconazole, but she died soon after.

**Conclusion:**

Our patient is the first case with isolated pulmonary valve endocarditis caused by *C. guilliermondii* in patients with uremia. Hematologic disorders, in addition to long-term central venous catheter use, prolonged antibiotic intravenous injection, and congenital cardiac anomaly, predispose to the condition. The diagnosis “isolated” pulmonary IE is difficult, and combing surgery with antifungal antibiotics is the appropriate therapeutic management for Candida related pulmonary IE.

## Background

Right-sided infective endocarditis (IE) is uncommon compared with left-sided IE, representing only 5–10% of all IE cases. Isolated pulmonary valve endocarditis without involvement of another valve constitutes <2% of IE cases, whereas fungal endocarditis comprises less than 10% [1, 2]. Isolated pulmonary valve IE caused by *Candida* is rare, and nonsurgical treatment— though not recommended by the 2009 Infectious Diseases Society of America candidiasis guidelines for Candida endocarditis (which suggest combining surgery with antibiotics) [3]—has been reported to be successful [4]. We present the case of a chronic HD patient who presented with isolated pulmonary valve IE caused by non-*albicans Candida*, and we review the literature on isolated pulmonary valve IE caused by *Candida* spp.

## Case presentation

**Fig. 1:**
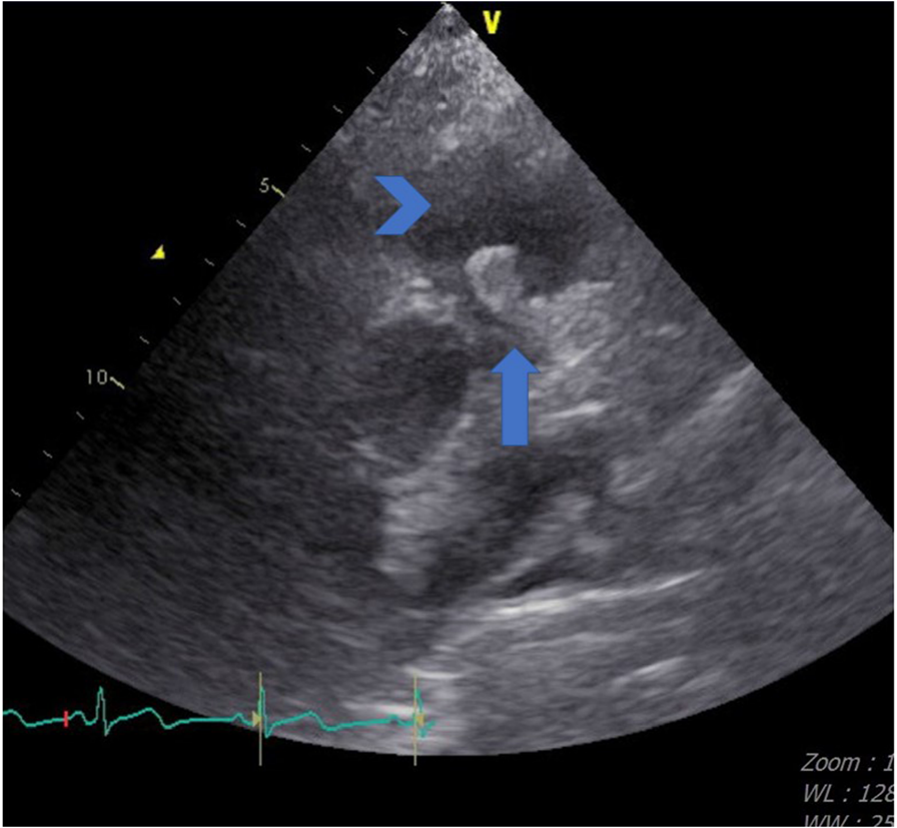
Vegetation on the pulmonary valve (arrow) near the pulmonary artery (arrowhead) on August 21, 2015. The size of vegetation was 1.2 cm

**Fig. 2:**
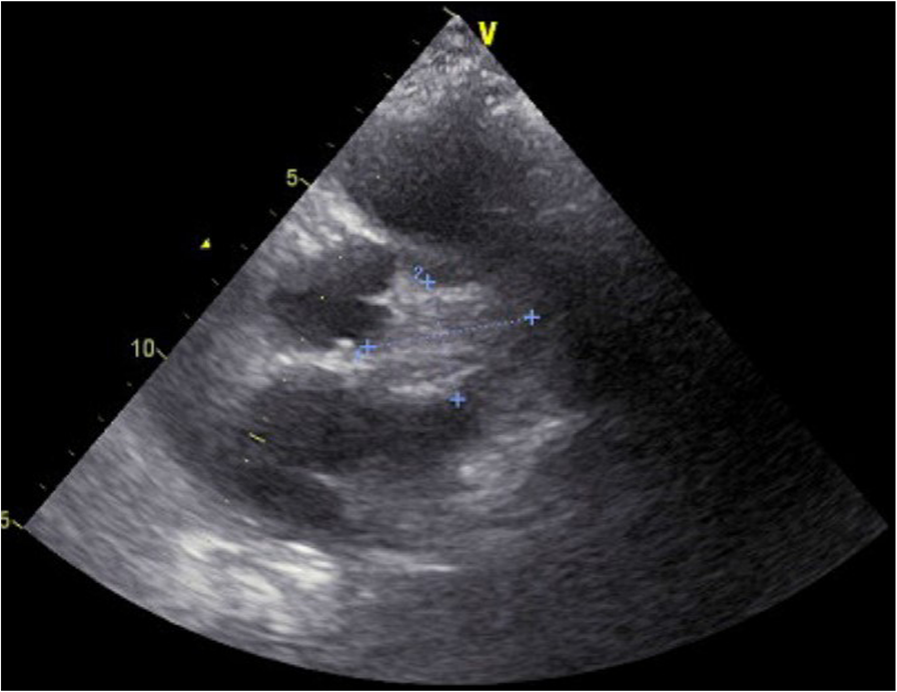
Vegetation on the pulmonary valve on September 17, 2015, revealing growth (3.37 × 2.70 cm)

**Fig. 3:**
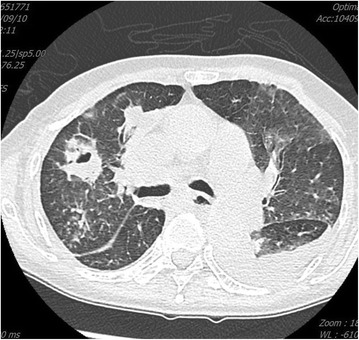
Computed tomography revealing right upper-lung pneumonia, the air–fluid level, and septic embolism

**Fig. 4:**
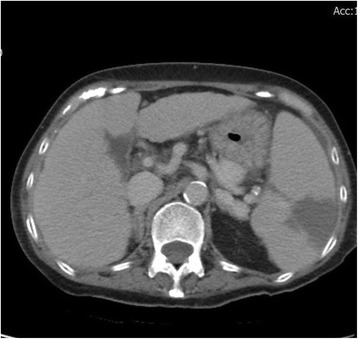
Computed tomography of the abdomen, revealing a splenic infarction

A 63-year-old woman with a history of ESRD was admitted to our hospital on June 3, 2015 after experiencing disorientation for 5 days. The patient had been receiving HD since December 2014 because of acute on chronic kidney disease due to pneumonia. She was also diagnosed with hepatitis B-related liver cirrhosis (Child-Pugh B with hepatic encephalopathy), *Mycobacterium tuberculosis*-related pleuritis, and IgGλ monoclonal gammopathy. Monoclonal gammapathy hadn’t been treated because she was permanently bedridden. She received maintenance HD through a tunneled-cuffed catheter inserted into the right subclavian vein since December 8th, 2014. She denied active intravenous drug use. We observed drowsy consciousness and splenomegaly during physical examination. No crackles were found in either lung field, and no track marks were present on her skin. Her white blood cell, absolute neutrophil, and platelet counts were 2.86 × 10^3^/uL, 2116/mm^3^, and 14,000/uL, respectively. Her total bilirubin was 3.54 mg/dL. In addition, C-reactive protein was 2.31 mg/dL, and her serum glucose was 992 mg/dL, without metabolic acidosis. Because of the hyperglycemic hyperosmotic status of the patient, blood culture was drawn and empiric vancomycin and cefuroxime were prescribed. The initial blood culture grew *Candida guilliermondii* without other bacteria. Fluconazole 200 mg once per day was administered intravenously. The tunneled-cuffed catheter was removed on June 30 because of persistent fungemia. The culture of tunneled-cuffed catheter grew *Candida guilliermondii.*. Blood cultures on July 14 and August 10 and 28 still grew *C. guilliermondii*, even after replacement of caspofungin by fluconazole on July 28. No positive culture result was found in sputum or urine during the 8 weeks after admission. Transthoracic echocardiography on July 20 and August 21 revealed no vegetation or congenital abnormality. On August 25, repeated transthoracic echocardiograms showed a 1.2-cm mobile mass on the pulmonary valve extending from the right ventricular inflow tract across the pulmonary valve (Figs. 1 and 2). No other vegetation was found. Surgical intervention was suggested, but the family of the patient declined because of her multiple comorbidities. Therefore, amphotericin B 40 mg was administered once daily from August 30 but was discontinued on September 1 because of allergic reactions (rash and fever). After 8 weeks of caspofungin, the *C. guilliermondii* septicemia was still present and the vegetation on the pulmonary valve had increased in size (3.73 × 2.70 cm). Computed tomography (CT) of the chest and abdomen revealed splenic infarction and right upper lung pneumonia with septic embolism (Figs. 3 and 4). The patient and family requested hospice care, and we discharged the patient with a long-term prescription of fluconazole 200 mg/d. The patient died from hepatic encephalopathy and coma on September 26, 2015.

## Discussion

This is the first reported case of non-albican Candida related isolated pulmonary valve endocarditis in a chronic HD patient. *Candida* spp.-associated bloodstream infection has become more prevalent in recent decades because of the increasing incidence of immunodeficiency (e.g., HIV infection, chemotherapy, and immunosuppressant use) and diverse invasive procedures (e.g., central venous catheters and intracardiac devices) [2]. In intensive care unit patients with ESRD, central venous catheterization was the only risk factor significantly associated with candida-related blood stream infection [5]. Among *Candida*-caused invasive infections, *C. guilliermondii* is rare (0.4–1.4%), and in vitro susceptibility to fluconazole and voriconazole is 75.7 and 91.7%, respectively [6]. The percentage of *C. guilliermondii* in total Candida species is 1.1% in the Asian-Pacific region, and fluconazole susceptibility is 77.4% [6]. The specimen type that most commonly yields *C. guilliermondii* is blood, followed by skin and soft tissue [5, 7]. *C. guilliermondii*-caused infections are rare in patients with hematologic malignancies, solid tumors, or neutropenia and concomitant bacterial infection [7]. The mechanism of immunity to non-*albicans Candida* species is not well understood; however, it has been observed that the antigenicity of *C. guilliermondii* differs from that of *Candida albicans*, and that the pattern recognition receptor for *C. albicans*—such as galectin-3—does not affect *C. guilliermondii*. *C. guilliermondii* also secretes serine proteinase, which hydrolyzes extracellular protein and destroys a broad spectrum of relevant host proteins [8].

Isolated pulmonary valve endocarditis is rare, and the most common pathogen for the condition is *Staphylococcus aureus*. A lower pressure gradient leading to the pulmonic valve receiving less shear stress compared with the other valves has been proposed as a possible mechanism for the condition, whereas underlying congenital or acquired valvular abnormality involving pulmonary valves has been suggested as a less common mechanism [8]. Congenital heart disease is commonly mentioned in the literature on isolated pulmonary valve endocarditis [9]. The use of a central venous catheter, which is becoming more common, is a risk factor for health care-associated IE. Right-sided IE occurs in 5-10% of all IE patients, and isolated pulmonary valve involvement is present in 2.5% of IE cases. In our patient, the vegetation developed slowly (more than 2 months after admission); diagnosis for isolated pulmonary valve endocarditis is difficult [10].

Table 1 presents a summary of literature found using MEDLINE and PubMed on isolated pulmonary endocarditis caused by *Candida* or another fungus. There have been only 3 similar published cases. The data indicate that the condition is predominantly found in males and caused by Candida. In addition to congenital cardiac anomaly and prolonged intravenous drug infusion, hematologic disorders, such as transient neutropenia [8] and bone marrow infection caused by protozoa [11], are possible risk factors. Our patient had multiple risk factors such as monoclonal gammopathy and long-term catheter use. Dyspnea and respiratory failure caused by pulmonary embolism [4] is common in patients with isolated pulmonary IE. However, in the current case, splenic infarction was observed during admission, and the patient didn’t need additional oxygen supply. The most widely recommended management method of isolated pulmonary valve *Candida*-associated endocarditis is pulmonary valve resection with prolonged antifungal antibiotics use. The American College of Cardiology and the American Heart Association guidelines list a Class 1 recommendation that fungal endocarditis be considered an indication for surgery; however, *Candida*-associated IE commonly occurs in patients who are poor surgical candidates because they have multiple comorbidities [12]. Although Devathi et al. treated a patient with antifungal antibiotics successfully without surgery, this approach was unsuccessful in our patient. Therefore, in patients with isolated pulmonary valve IE caused by *Candida*, antibiotics alone may be insufficient for treating patients with isolated pulmonary IE, and surgical resection should be mandated as the curative treatment.

**Table 1:** Literatures about Candida- associated pulmonary valve endocarditis

	Patient (age/gender)	Risk Factor	Presentation	Candida	Surgical management	Antibiotics use and duration	Outcome
Devathi et al. [4]	61/ male	1. Intravenous drug abuser. 2. Transient neutropenia	Hypoxemic respiratory failure; pulmonary valve vegetation 1.5 cm.	*Candida albicans*	Not performed	Liposomal amphotericin B for 8 weeks	No recurrence in 6 months.
Uchida et al. [13]	66/ male	*Staphylococcus aureus* sepsis with exposure to broad spectrum antibiotics	Multifocal pulmonary embolism and severe pulmonary regurgitation.	*Candida parapsilosis*	Resection of pulmonary valve without replacement.	Amphotericin B for 8 weeks.	Severe pulmonary regurgitation 2 years after operation.
Darwanzah et al. [11]	17/ male	1. Patent Ductus arteriosus. 2. Visceral Leishmaniasis in bone marrow. 3. Prolonged intravenous injection of antibiotics and fluid.	1. Congestive heart failure 2. Acute renal failure with HD. 3. Pulmonary valve vegetation 0.9 cm.	*Candida albicans*	1. Resection of pulmonary valve with repairment. 2. Ligation of PDA.	Amphotericin B	No recurrence in 2 years.
Hou et al.	63/ female	1. Tunneled-cuff catheter 2. Monoclonal gammapathy 3. Chronic HD. 4. Liver cirrhosis	1. Persistent fungemia 2. Splenic infarction.	*Candida guilliermondii*	N/A	Fluconazole for 8 weeks. Caspofungin for 8 weeks followed by fluconazole 200 mg daily.	Expired

## Conclusion

Isolated pulmonary valve endocarditis caused by *C. guilliermondii* is rare. Hematologic disorders, in addition to long-term catheter use, prolonged intravenous injection, and congenital cardiac anomaly, predispose patients to the condition. The diagnosis for isolated pulmonary IE is difficult, and combing surgery with antifungal antibiotics is the appropriate therapeutic management for Candida related pulmonary IE.

## Abbreviation

CT: Computed tomography
ESRD: End stage renal disease
HD: Hemodialysis
HIV: Human immunodeficiency virus
IE: Infective endocarditis
